# Untreated Depression During Pregnancy and Its Effect on Pregnancy Outcomes: A Systematic Review

**DOI:** 10.7759/cureus.17251

**Published:** 2021-08-17

**Authors:** Nasrin Jahan, Terry R Went, Waleed Sultan, Alisha Sapkota, Hajra Khurshid, Israa A Qureshi, Michael Alfonso

**Affiliations:** 1 Psychiatry, California Institute of Behavioral Neurosciences & Psychology, Fairfield, USA; 2 Medicine, California Institute of Behavioral Neurosciences & Psychology, Fairfield, USA; 3 Medicine, Beni Suef University Faculty of Medicine, Beni Suef, EGY; 4 Surgery, Halifax Health Medical Center, Daytona Beach, USA; 5 Internal Medicine, California Institute of Behavioral Neurosciences & Psychology, Fairfield, USA; 6 School of Medicine, Universidad del Rosario, Bogota, COL

**Keywords:** untreated depression, maternal depression, pregnancy outcomes, maternal outcomes, neonatal outcomes

## Abstract

Depression is characterized by sad, irritated, or empty moods, as well as somatic and cognitive changes such as loss of concentration, anhedonia, hopelessness, loss of appetite, sleep disturbances, and suicidal ideation, all of which have a negative impact on an individual's ability to function. Depression that occurs during pregnancy is known as antenatal depression. The occurrence of depression during pregnancy and afterward is quite high. Women having a history of depression before pregnancy have a high probability of getting depression during pregnancy again. The purpose of the study is to review the effect of untreated depression during pregnancy on maternal and neonatal outcomes. The primary outcomes of this review were the identification of studies showing the relationship between untreated depression during the pregnancy indicated by depression measures and any associated adverse birth outcomes; specifically, low birth weight, small for gestational age, preterm birth, postpartum depression, and infant neurodevelopmental outcome. We reviewed 20 population-based contemporary cohort studies with a range of populations from 54 to 194,494, all of them representing the population of gestational age located in multiple jurisdictions. It was found that maternal depression during pregnancy has a positive association with preterm birth, small for gestational age, stillbirth, low birth weight, and maternal morbidity including perinatal complications, increased operative delivery, and postpartum depression. To prevent these adverse outcomes, depression should be screened, monitored, and managed appropriately keeping risk-benefit in consideration.

## Introduction and background

Depression is defined as a state of sadness, irritability, or empty moods, as well as somatic and cognitive changes such as loss of concentration, anhedonia (lack of pleasure), hopelessness, loss of appetite, sleep disturbances, and suicidal ideation, all of which have a negative impact on an individual's ability to function. Depression that occurs during pregnancy is called prenatal or antenatal depression. Prenatal depression should be identified and treated like postpartum depression. However, prenatal depression is repeatedly not considered a vital health issue. According to the American College of Obstetricians and Gynecologists, clinicians should screen for prenatal depression with a standard tool at least once during pregnancy. Despite a patient having been diagnosed with depression, treatment is often declined because of concerns regarding the baby’s health. Although there are several studies on prenatal depression, the research results of the depression on the pregnancy outcomes were unpredictable. Prenatal depression has been linked to preterm delivery, low birth weight, and intrauterine growth restriction in some studies, whereas depression has been found to not affect pregnancy outcomes such as preterm birth, low birth weight, and poor Apgar scores in others [1].

Based on the current Diagnostic and Statistical Manual of Mental Disorders, 5th edition (DSM5), major depression is diagnosed by the presence of five out of nine stated symptoms within two weeks and having a considerable negative influence on the previous functioning [2]. It mentions that the symptoms should not be attributable to another medical condition and, in this case, pregnancy. Antenatal depression falls under the specifier termed "with peripartum onset." The mood episodes may present with or without psychotic features. Diagnostic criteria consist of depressed mood, considerably lessened interest or pleasure in most activities, psychomotor retardation, or agitation, feeling of worthlessness or inappropriate guilt, impaired thinking or concentration, and repeated thoughts of death [2]. Somatic symptoms including significant weight and appetite variations, and changes in sleep patterns and fatigue, may also appear in any normal pregnancy due to neuroendocrine physiological changes [3].

Major depressive disorder, also known as clinical depression, is common in women of childbearing age [4]. The prevalence of postpartum depression is 17%, while the incidence of prenatal depression is as high as 20% to 40%. Depression rates during pregnancy have been reported to be as high as 7.4% in the first trimester, 12.0-12.8% in the second and third trimesters, and even higher rates in the first year after delivery [1]. Women with a history of depression before pregnancy are especially at risk with high depression relapse rates during pregnancy [4].

There are several risk factors related to the increased risk of developing depressive symptoms during pregnancy that has been studied particularly in low- and middle-income countries. A study performed by Howard et al. (2013) revealed that intimate partner violence was significantly coupled with depression during pregnancy [2]. On the other hand, Fischer et al. (2012) found that life stress and major adverse life events, poor socioeconomic status, absence of perceived social or relationship support, unwanted or unintended pregnancies, and the decision made by some women or their clinicians to stop antidepressant maintenance medications were a key risk factor in increasing depression in the antenatal period [2]. Prior history of psychopathology that is largely related to depression, preconception anxiety, and young age is also correlated with the risk of developing clinical depression during pregnancy. Other elements thought to predispose to depression in the general population include genetic and hormonal susceptibility, chronic illness e.g., acquired immunodeficiency syndrome (AIDS), and personality traits [2]. 

Depression can have major repercussions for both the mother and the fetus if it occurs during pregnancy. Some of those consequences linked to prenatal depression are slower fetal development, abortion, low birth weight, preterm labor, preterm birth, maternal anemia and diabetes, hypertensive disorders (including preeclampsia), cesarean section (CS), and postpartum depression. Infants born to mothers who are depressed during pregnancy are more irritable, less active, and more likely to experience developmental delays. Prenatal depression also raises the risk of postpartum depression, and the infant's health and growth may be harmed if the mother is depressed for an extended period. Despite the severe consequences of prenatal depression and the possibility that proper screening and treatment may help to reduce adverse birth outcomes, most research has concentrated exclusively on postpartum depression [5].

Pregnancy is a significant driver of antidepressant drug discontinuation, and most women do not get additional antidepressant prescriptions after six weeks of pregnancy. Despite the significance of depression, screening for it is neglected, avoiding treatment and measures to prevent the escalation of symptoms and their effects; then, oftentimes when depression is identified, it is frequently left untreated [5]. Even though women should be screened and treated before conception, many women go into pregnancy with untreated depression [6]. Hence, this study aims to review the effect of untreated depression during pregnancy on maternal and neonatal outcomes.

## Review

Methods

Literature Search

We followed the Preferred Reporting Items for Systematic Reviews and Meta-Analyses (PRISMA) guidelines for this systematic review. We conducted a systematic literature review where two authors (JN and WT) independently searched Medline in PubMed, PubMed Central (PMC), and Google Scholar databases for relevant articles from January 2014 up to April 2021, and additional papers were retrieved due to relevance. After this process, duplicates were removed, and manual screening was performed to select relevant studies.

The primary outcomes of this review were identification of studies indicating the relationship between untreated depression during the pregnancy indicated by depression measures and any associated adverse birth outcomes; specifically, preterm birth, low birth weight, small for gestational age, postpartum depression, and infant neurodevelopmental outcomes such as autism spectrum disorder (ASD), attention deficit hyperactivity disorder (ADHD), anxiety, and depression. Additional new studies were identified manually using citation searches based on their relevancy.

Inclusion Criteria

Papers fulfilling following criteria were included during the literature search from database and registry: (a) results retrieved based on a literature search of English language articles only; (b) only included studies published between January 1st, 2014 and April 30th, 2021; (c) full text papers; (d) studies conducted on humans; (e) women of childbearing age between 14 and 45 years; (f) only articles clearly focused on untreated gestational depression; (g) studies where depression/depressive symptoms were included as a primary independent variable and preterm birth (PTB), low birth weight (LBW), small for gestational age (SGA), spontaneous abortion, maternal effects including preeclampsia, cesarean section (CS), operative vaginal delivery, increase use of epidural analgesia during delivery, suicidal ideation, and the development of postnatal depression were consequence of primary independent variable; (h) population-based, cohort, and case control studies; (i) studies that used self-reported questionnaires or organized psychiatric interviews to assess depression, anxiety, and stress symptoms in all pregnant women; and (j) articles in which proven diagnostic or screening techniques were used to detect depression.

Exclusion Criteria

Studies with the following criteria were excluded: (a) non-human studies; (b) study focused on stress and anxiety; (c) the use of antidepressant medication was the focus, rather than the measurement and diagnosis of depression; (d) patients that reported use of antidepressant during pregnancy also were excluded; and (e) articles in languages other than English.

Search Strategy

The keywords used during the search were "depression during pregnancy," "pregnancy outcomes," "perinatal outcome," "preterm birth," "neonatal outcome," and "female."

A methodical search of the databases mentioned was conducted on May 1, 2021. The search for relevant studies using generic keywords and MeSH terms was the following: “depression during pregnancy” AND “pregnancy outcomes” OR “perinatal outcome” OR “preterm birth” OR “neonatal outcome” AND “female.” Periods between January 1st, 2014, and April 30th, 2021, were addressed. A total of 1,225 studies were identified. After removing 38 duplicate articles, 1187 unique articles were identified.

Screening

We evaluated the titles of all the citations received from electronic database searches for the first stage of screening and eliminated any that were not focused on untreated gestational depression. The abstract review of accessible publications (either free or through access privilege as a member of an educational institution) was the second stage of screening, and full-text inclusion and exclusion criteria were applied. Such screening resulted in the identification of 24 articles. Full-text articles were obtained for these screened abstracts except for three of them which were not accessible. In addition, two new studies were identified via citation search where inclusion and exclusion criteria were met. Full-text articles were accessed for all of them.

Critical Appraisal of the Literature

The study quality was assessed independently by two investigators (JN and WT) for all 23 articles screened and accessed. All of them are cohort study while one of them is a nested case-control study. We used the Newcastle-Ottawa Scale (NOS) for the assessment of study quality. The total NOS score was 9, which was suitable for evaluating case-control studies (selection, comparability, and exposure) and cohort studies (selection, comparability, and outcome). These studies were deemed to be high quality, with all studies having at least a 7 NOS score.

Results

**Figure 1:**
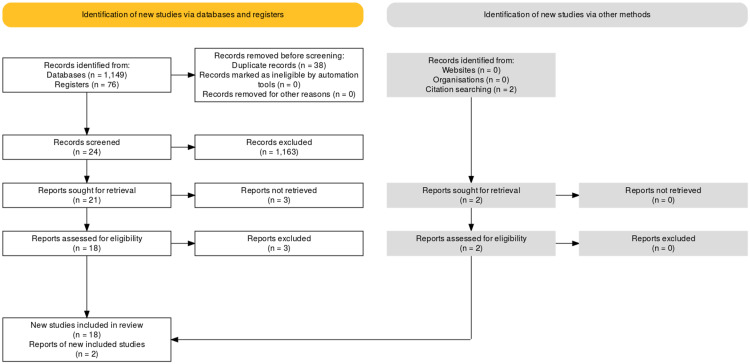
PRISMA 2020 flow diagram PRISMA, Preferred Reporting Items for Systematic Reviews and Meta-Analyses.

Our search in databases and registers resulted in 1,225 articles. After removing 38 duplicates, we went through titles and abstracts obtaining 24 articles. We could access 21 of them, while the other three were not accessible, so we excluded those papers. After going over full-text papers, we excluded three more papers as they were not addressing our research question. In addition, we identified two more articles through citation search. We selected 18 articles plus two additional articles, which were assessed for the quality of the studies by using NOS. All 20 articles were cohort studies. Brief details of these steps are available in the flow diagram below (Figure 1) [7].

Identification of Studies via Database and Register

All these 20 studies were population-based cohort studies with a range of population from 54 to 194,494, representing the population of gestational age located in multiple jurisdictions. Study characteristics, i.e., data collection method and study sets, differ, but in common, all these studies utilized data that were collected from individual subjects. Most of the studies measured depression during pregnancy using the Edinburgh Postnatal Depressive Scale (EPDS), while few others relied on the Center for Epidemiological Studies-Depression (CES-D), Patient Health Questionnaire-9 (PHQ9), Beck Anxiety Inventory (BAI), and custom questionnaires. These studies exhibit information regarding the association of gestational depression with the impact it has over a wide range of outcomes, making a special emphasis on low birth weight, preterm birth, postpartum depression, and infant neurodevelopmental outcome. We tabulated these 20 articles to evaluate the indicated association between untreated depression during the pregnancy in the context of proper depression measures and any associated adverse birth outcomes. A summary is available in Table 1 as follows:

**Table 1:** Association of depression during pregnancy and pregnancy outcomes SGA = small for gestational age; ASD = autism spectrum disorder; PTB = preterm birth; LGA = large for gestational age; CS = cesarean section; IUGR = intrauterine growth retardation; SCN = special care nursery; NICU = neonatal intensive care unit; PHQ9 = Patient Health Questionnaire-9; DSM-IV = Diagnostic and Statistical Manual of Mental Disorders, Edition 4; DADP = depression and anxiety during pregnancy; CES-D = Center for Epidemiological Studies-Depression; EDPS = Edinburgh Postnatal Depression Scale.

Authors and year	Study types	Purpose of study	Study characteristics	Sample size	Depression measures	Actual result	Conclusion
Weobong et al. (2014) [8]	Cohort study	To investigate the effects of depression on a baby's survival	To identify pregnancies and collect data on births and deaths, a cohort study was placed within four-weekly surveillance of all women of reproductive age	n = 20,679	PHQ9 to ascertain and DSM-IV to determine major or minor depression	Premature labor, peripartum difficulties, postpartum complications, non-vaginal delivery, infant sickness, and bed net use during pregnancy were found linked, but neonatal fatalities, stillbirths, low birth weight, immediate breastfeeding initiation, or exclusive breastfeeding did not indicate any link	This leads to premature labor, peripartum difficulties, postpartum complications, non-vaginal delivery, infant sickness, and bed net use during pregnancy
Xu et al. (2020) [1]	Cohort study	To see if prenatal depression throughout late pregnancy has an impact on maternal and infant outcomes	Clinical data of subjects were divided into three groups: mild-to-moderate depression, severe depression, and non-depression	n = 595	Edinburgh Postnatal Depressive Scale (EPDS)	Pregnant patients with severe depression were more likely to have preterm delivery than women without depression or with mild-to-moderate depression	Preterm birth is substantially linked to severe prenatal depression throughout late pregnancy, whereas mild-to-moderate prenatal depression had little effect on mother or neonatal outcomes
Mochache et al. (2018) [3]	Cohort study	The goal of this study was to see if prenatal depression is linked to preterm birth	Even though 292 women were recruited, only 255 were successfully followed up to delivery, owing to a 12.7% attrition rate	n = 292	Edinburgh Postnatal Depression Scale (EPDS)	Those with depressive symptoms had a 3.8 times increased risk of giving birth prematurely	There was a link between prenatal depression and premature labor and delivery
Szegda et al. (2017) [4]	Cohort study	To look at the associations between depression, preterm birth, and small for gestational age	Early, mid, and late pregnancy interviews were conducted	n = 1,262	Edinburgh Postnatal Depression Scale (EPDS)	Mid-pregnancy depression increased the risk for SGA. SGA was not associated with late pregnancy depression; preterm birth was not associated with depression during pregnancy	Mid-pregnancy depression increased the incidence of SGA in this cohort of mostly Puerto Rican women
Fekadu Dadi et al. (2020) [9]	Cohort study	To determine the prevalence of unfavorable birth outcomes, as well as the direct and indirect pathways through which depression and other psychosocial risk factors may influence these outcomes	Women in their second or third trimester were followed for a month after giving birth	n = 916	Edinburgh Postnatal Depression Scale (EPDS)	In general, there was no association between antenatal depression and preterm birth. However, depression was modified by partner support and stress coping ability, and preterm birth was 4.38 and 4.99 times higher for those with poor spouse support and poor stress coping ability	No association is indicated between antenatal depression and preterm birth
Choi et al. (2014) [10]	Cohort study	To see if antenatal depressed symptoms have an impact on obstetric outcomes and if there are any links between antenatal and postpartum depression	Pregnant women in their third trimester of pregnancy who were getting obstetrical care; participants were split into two groups based on EPDS scores: non-depressed (n = 344, EPDS score 9) and depressed (n = 123, EPDS level 10)	n = 467	Edinburgh Postnatal Depression Scale (EPDS)	With EPDS scores of ≥10, 26.34% (n = 123) of the 467 patients exhibited prenatal depressive symptoms. Prenatal depression symptoms were not linked to any significant perinatal consequences. A total of 192 women from the initial study cohort were given the EPDS again as a follow-up during the postpartum period. A total of 56 (29.17%) of the 192 participants received a score of 10 or higher. The prenatal and postpartum EPDS scores had a Spearman correlation coefficient of 0.604, which was statistically significant (P = 0.001)	Antenatal depression is not associated with poor perinatal outcomes. Screening for antenatal depression, on the other hand, may be useful in identifying women at risk of postpartum depression
Hagberg et al. (2018) [11]	Cohort study with nested sibling case-control analysis	To estimate the risk of ASD in children of women who had depression during pregnancy and use antidepressants compared to those who were not	Mother–baby pairings with a 12-month history of depression before delivery and a three-year follow-up period for the kid	n = 194,494	Known case of depression population	According to the findings, women who experience depression during pregnancy have a higher probability of having a kid with ASD than women who are not exposed	Women who are depressed during pregnancy have a higher risk of having a child with ASD, regardless of whether they take antidepressants
Benatar et al. (2020) [5]	Cohort study	To find out how common depression is among pregnant Medicaid recipients and what the link is between depression and pregnancy outcomes	From 2013 to 2017, Medicaid individuals registered in Strong Start for Mothers and Newborns 2, a national preterm birth prevention program, with a single pregnancy and reliable depression data	n = 37,287	Depression screening (a shortened version of the Center for Epidemiological Studies – Depression [CES-D] scale)	Women who screen positive for depression have a 2.5% increased chance of having a preterm birth (12.5% vs. 10.0%), a 2.4 percentage point higher probability of their infant being low birth weight (10.9% vs. 8.4%), and a 2.6 percentage point higher probability of C-section birth (28.8% vs. 26.3%) than women who are not depressed	Depression is positively correlated to preterm birth, low birth weight, and probability of C-section
Flynn et al. (2015) [12]	Cohort study	To find the effect of prenatal depression and anxiety on key infant outcomes (gestational age at birth, birth weight, and Apgar scores), and maternal medical conditions	Women who were presenting consecutively for prenatal care at a health system serving primarily Medicaid patients and information on maternal characteristics, maternal medical health as well as mental health were extracted	n = 419	Patient Health Questionnaire-9 (PHQ-9) and obstetrics clinician documentation of depression or anxiety	This study suggests that the interaction of prenatal depression and anxiety with medical conditions may have a greater impact on birth weight and gestational age	Prenatal depression and anxiety affect birth weight and gestational age
Yang et al. (2017) [13]	A population-based cohort study was used to create a nested case-control study	To find the effect of depression, anxiety, and both on low birth weight (LBW); and to examine if preterm birth (PTB) moderates these associations	The study used the Electronic Perinatal Health Care Information System to extract cases and controls	n (cases) = 5,457; n (control) = 2,853	Custom questionnaire	Depression and anxiety during pregnancy (DADP) are related to a greater incidence of LBW, with PTBs having the strongest association	The presence of DADP was associated with an increased risk of LBW
Nutor et al. (2018) [14]	Retrospective cohort study	The goal of this study was to look at the relationship between depressive symptoms and preterm birth while controlling for social support, both general and from the baby's father	Women were interviewed during their postpartum hospitalization 24 to 48 hours after birth. Medical records were used to obtain information about the neonates and their mothers' health	n = 1,207	Center for Epidemiological Studies-Depression (CES-D); CES-D scores ≥23 considered severe	Around 17% of the women in the sample had a PTB, while 20% of the women in the sample had a CES-D score of ≥23. After adjusting for both general social support and father of the baby support, women with a CES-D score of >23 were nearly 70% more likely to have a PTB than women with a CES-D score of <23 (PR = 1.68, 95% CI: 1.24-2.16)	Women with severe depression were almost twice more likely to have PTB compared with women with not severe depression
Smith et al. (2015) [15]	Retrospective cohort study	To investigate whether maternal depression during pregnancy affected adverse birth outcomes, specifically preterm birth and small for gestational age (SGA)	Used the Utah Pregnancy Risk Assessment Monitoring System, which is an ongoing surveillance initiative that analyzes maternal behaviors in women who have recently given birth	n = 4,123	Custom questionnaire	Women who were told they had depression by a doctor, nurse, or other health care worker during pregnancy had statistically significantly increased odds of preterm birth as compared to women who were not told they had depression. However, after adjustment for prenatal care visits, the depression–preterm birth association was attenuated and no longer statistically significant	The findings of this study do not find the correlation between maternal depression and adverse birth outcomes
Li et al. (2020) [16]	Prospective cohort study	To examine the associations of antenatal depression symptoms with pregnancy outcomes, especially for low birth weight	Unfavorable outcomes in pregnant women with prenatal depression were calculated using multivariate logistic regression (CI) that represented as odds ratio (OR) and 95% confidence interval (CI)	n = 1,377	The Edinburgh Postnatal Depression Scale (EPDS) questionnaire was used to assess depressive symptoms in the second trimester of pregnancy; EPDS ≧ 12 scores were included	After adjusting for maternal age, education, parity, pre-pregnancy BMI, residential region, and fetal gender, an EPDS score of ≥12 (vs. <12) was attributed to an elevated risk of low birth weight but not preterm birth, large for gestational age, small for gestational age, or macrosomia	Low birth weight is linked to maternal depression, although there is no link to preterm birth, SGA, LGA, or macrosomia
Rawahi et al. (2020) [17]	Prospective cohort study	To determine if there is a relationship between antenatal depression and pregnancy outcomes, including the risk of postpartum depression	Women with a gestational age of ≥32 weeks who were receiving antenatal care at 12 local health centers in Muscat, Oman, were invited to participate	n = 959	The Edinburgh Postnatal Depression Scale (EPDS)	Antenatal depression was associated with a greater risk of CS and postnatal depression, according to a logistic multivariate regression study	An increased risk of CS and postnatal depression is linked to antenatal depression
Eastwood et al. (2017) [18]	Retrospective cohort study	To look at the association between maternal depressive symptoms and key perinatal outcomes including birth weight, gestational age at birth, breastfeeding indicators, and postnatal depressive symptoms during pregnancy	Women were identified from routinely recorded antenatal data and determined the risk of adverse perinatal outcomes associated with maternal depression during pregnancy. Logistic regression models that adjusted for confounders were used to determine the result	n = 17,564	Edinburgh Postnatal Depression Scale (EPDS)	The cohort had a 7.0% prevalence of maternal depressive symptoms during pregnancy, which was strongly associated with postnatal depressive symptoms. Depressive symptoms before conception were associated with a higher odds of low birth weight and preterm birth compared to women who reported lower EPDS scores in the antenatal period. In the early postnatal period, antenatal depression symptoms were not strongly linked to non-exclusive breastfeeding	Prenatal maternal depressive symptoms are closely linked to postnatal depressive symptoms and negative perinatal outcomes
Sabri et al. (2015) [19]	Prospective cohort study	To assess the risk of preterm delivery and IUGR related to antenatal anxiety and depression throughout early pregnancy, as well as to assess their impact on fetal growth and birth outcome	In the late second trimester, the mother's sociodemographic data, EPDS, and the Beck Anxiety Inventory (BAI) were examined; fetal growth and activity were assessed during the ultrasound examination, as well as the doppler waveforms investigation of the umbilical vessels after 30 weeks of pregnancy	n = 54	Edinburgh Postnatal Depressive Scale (EPDS) and the Beck Anxiety Inventory (BAI)	Women experiencing depressive and anxiety symptoms in the third trimester of pregnancy are more likely to have oligohydramnios, IUGR, decreased placental perfusion, and preterm labor, according to this study	Various fetal developmental abnormalities have been related to maternal depression and anxiety symptoms during pregnancy
Uguz et al. (2019) [20]	Cross-sectional study design	To see how maternal major depression, anxiety disorders, and their comorbidities affect infants' gestational age and birth weight	The study comprised 1119 women, including 26 women having only major depression, 125 women having an only anxiety disorder, 36 women having severe depression plus anxiety disorder, and 932 women having no mental problems	n = 1,119	Structured psychiatric interviews were conducted using the Structured Clinical Interview for the Diagnostic and Statistical Manual of Mental Disorders, Fourth Edition (DSM-IV) (SCID-I)	The comorbid group had the highest percentage of babies who were born prematurely or with low birth weight	During pregnancy, comorbidity between serious depression and anxiety disorders may have significant unfavorable consequences on birth weight and gestational age
Khanghah et al., 2020 [21]	Cohort study	The goal of this study was to see if there was an association between antenatal depression, pregnancy, and neonatal outcomes	This study used a convenient sample approach and collected data using questionnaires on demographic and obstetric variables, the CES-D depression scale, and a pregnancy outcome checklist	n = 394	Center for Epidemiological Studies-Depression (CES-D)	Preeclampsia, premature membrane rupture, preterm delivery, cesarean section, intrauterine fetal death, and intrauterine fetal growth restriction were higher among mothers with depression during their pregnancies. The mean birth weights of depressed mothers' infants were lower than the infants of mothers who did not have depression	Depression is associated with a worse pregnancy and neonatal outcome when it occurs during pregnancy
Dowse et al. (2020) [22]	Retrospective cohort study	To see if there is any connection between self-reported depression, self-reported anxiety, and neonatal birth outcomes using EPDS scores	Linear regression and logistic regression were used to examine the effect on birth weight, gestational age, admission to the NICU or the SCN, birth outcome (stillborn vs. live birth), and Apgar scores. The influence on the newborn length of stay was estimated using Cox proportional hazards regression	n = 53,646	The Edinburgh Postnatal Depression Scale	Women with self-reported anxiety were more likely to have birth complications, have more hospital admission, have worse Apgar ratings, and spend longer in the hospital. Babies born to women who self-identify as depressed are more likely to have a lower birth weight, shorter gestational age, and a worse Apgar score with a longer stay in the hospital	Lower birth weight, shorter gestational age, and lower Apgar score are all associated with maternal depression
Hermon et al. (2019) [23]	Cohort study	To estimate the risk of maternal depression among women hospitalized in a high-risk pregnancy department, and to evaluate its potential association with adverse perinatal outcome	During the study period, 279 women met the inclusion criteria. Among them, 28.3% (n = 79) screened positive for depression (≥10 points on the EPDS)	n = 279	The Edinburgh Postnatal Depression Scale (EPDS)	Preterm birth, low birth weight, low Apgar scores, and neonatal intensive care unit (NICU) admissions were all shown to be significantly higher in the screen-positive group in the univariate analysis. Maternal antenatal depression during hospitalization was found to be an independent risk factor for preterm delivery in a multivariate regression model that controlled for maternal age, ethnicity, gestational diabetes mellitus, preeclampsia, previous preterm delivery, and gestational age at admission	Maternal depression is associated with adverse perinatal outcomes

Discussion

Women's depression is a prevalent ailment, with rates rising around childbearing years. Any numbers between 10% and 23% of women, who are 18 or older, have experienced symptoms of depression, which often include sleeplessness, hopelessness, and low energy [24,25]. Moreover, when women are evaluated, 6.5% to 13% of women screen positive for prenatal depression and these rates are similar among pregnant women and comparable non-pregnant women [5,26].

There are concerns about the effects of antidepressant use during pregnancy on offspring. Antidepressant drugs' effects on growing fetuses are not well understood [15]. Antidepressants such as selective serotonin reuptake inhibitors (SSRIs) are widely administered during pregnancy. These medications have been shown to pass the placenta, and serotonin is essential for embryonic brain development [27]. Antidepressants are sometimes avoided by women who are pregnant or planning to get pregnant.

Between 1% and 13% of pregnant women receive treatment for depression during their pregnancy, as indicated by antidepressant prescriptions [11]. According to studies, women with a history of depression who stopped using antidepressants during pregnancy are more likely to relapse than those who keep taking them [5]. Discontinuation of any depression care is quite common during pregnancy. The rate of antidepressant prescription is approximate 70% during pre-pregnancy of depressed women. This rate drops to 27% during pregnancy [28]. Pregnancy is a significant driver of antidepressant drug discontinuation, and most women do not get additional antidepressant prescriptions after six weeks of pregnancy [5]. Despite depression’s seriousness, screening for depression is underutilized, which precludes treatment and efforts to prevent the escalation of symptoms and associated consequences. Even when depression is diagnosed, it often remains untreated [5]. Although women would normally be given screening and treatment before conception, many women go into pregnancy with untreated depression [5].

Depression Measures

Depression during pregnancy was the primary exposure variable in this study. Most of the studies screened and reviewed in this study were assessed using the Edinburgh Postnatal Depression Scale (EPDS) (studies in references [1,3,4,9,10,16-19,22,23]). However, the threshold for EPDS differs from study to study. It indicates that all these studies considered a minimum score of EPDS to determine gestational depression. Few others utilized other measures, i.e., the Center for Epidemiological Studies-Depression (CES-D) scale, the 9-item Patient Health Questionnaire (PHQ-9), and other custom questionnaires (studies in references [5,8,11-15,20,21]).

Factors Associated With Depression During Pregnancy

Various factors related to mental during pregnancy have been reported in these studies. According to Choi et al. (2014), the depressed group had more pregnant women with low levels of education than the nondepressed group. In addition, the depressed group had more unemployed women than the non-depressed group. The marital status of the two groups differed significantly, with the depressed group having more women who were single or had remarried than the nondepressed group. Women in extended families were more prevalent in the non-depressed group than in the depressed group [10].

Benatar et al. (2020) showed that black participants are considerably more likely to be depressed than white participants, while Hispanic participants are significantly less likely to be depressed. Women with a previous pregnancy are significantly more likely to be depressed than those without, as are women whose pregnancy is unintended compared with those who intended their pregnancy. Women who are married or in a relationship are much less likely to be depressed than those who are not married nor in a relationship. Having experienced intimate partner violence (IPV) is substantially associated with depression. Women who report challenges to attend antenatal appointments are substantially more likely to be depressed than women who report no barriers [5]. In addition, Li et al. (2020) reported that women under the age of 25 were more likely to suffer from antenatal depression than their counterparts [16].

Maternal Complications

According to Weobong et al. (2014), mothers with antenatal depression used to have a considerably higher risk of severe peripartum complications, postpartum problems, cesarean section and/or instrumental delivery, and prolonged labor. Heavy bleeding, a tear in the vaginal wall, placental abnormalities, and convulsions were four of the eight peripartum complications that depressive women were more likely to report antenatally. Further analysis showed that postpartum complications, such as fever and other major complications including leaking urine/feces, were substantially more common among antenatally depressed women. Additionally, it was revealed that women suffering from prenatal depression were much less likely to use a bed net during their pregnancy. According to their findings, there was no link between prenatal depression and antenatal care attendance, birth in a health facility, immediate breastfeeding initiation, or exclusive breastfeeding during the neonatal period [8].

According to Al Rawahi et al. (2020), there is a strong link between antenatal depression and CS delivery, which is supported by previous research. It was found that the women suffering from prenatal depression have higher levels of anxiety and fear of labor, resulting in lower pain tolerance, a higher need for epidural analgesia, and a higher rate of CS. Furthermore, psychological distress has been shown to impede uterine contractility, resulting in protracted labor, inability to progress, and eventually fetal distress, all of which could raise the chance of CS. [17]. Another study conducted by Khanghah et al. showed that preeclampsia, preterm rupture of the membranes, premature delivery, cesarean section, intrauterine fetal death, and intrauterine fetal growth limitation were all considerably greater among mothers with severe depression compared to those who did not have depression [21].

Perinatal Complications

In a study conducted in the United Kingdom, Eastwood et al. (2017) found that guilt was a major concern for depressive mothers who had breastfeeding difficulties, regardless of whether they continued nursing. This problem was linked to thoughts and sentiments of not being a perfect mother. It also showed that even after controlling for socioeconomic level, prenatal and postnatal depression are still significant contributors to poor baby feeding behaviors. Mother's mental and physical concerns, psychosocial factors (such as a lack of financial resources or inadequate family support), and reduced health-seeking behaviors are all plausible pathways for maternal depression's effect on infant feeding practices [18].

Fetal Outcomes

Benatar et al. (2020) show that women who screen positive for depression have a 2.5 percentage point higher rate of preterm birth, a 2.4 percentage point higher probability of their infant being low birth weight, and a 2.6 percentage point higher probability of C-section birth than women who are not depressed. Women who screen positive for depression are also 3.7 percentage points less likely to report initiating breastfeeding in the postpartum than other women [5].

According to Fekadu Dadi et al., although there is no direct link between prenatal depression and preterm birth, their study revealed that preterm birth was 4.38 and 4.99 times more common in individuals with poor spouse support and poor stress coping ability than in those with good spouse support and good stress coping ability, respectively. In the path analysis, depression had a significant indirect effect on preterm birth via partner support and delivery fear, both of which had independent direct effects on preterm birth [9].

**Figure 2:**
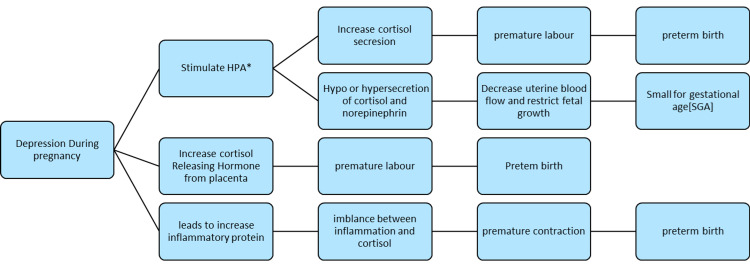
Effect of depression during pregnancy on fetal outcomes *HPA: hypothalamus-pituitary-adrenal.

Despite the uneven link between maternal depression and delivery outcomes, a biologically feasible explanation exists, and according to Smith et al. (2015) and Fekadu Dadi et al. (2020), researchers have found that depression stimulates the hypothalamus-pituitary-adrenal (HPA) system during pregnancy, resulting in increased cortisol hormone output. The placental clock is a scenario in which depression during pregnancy stimulates the synthesis of corticotrophin-releasing hormone (CRH) from the placenta, causing preterm labor. Chronic stress can also impair the body's ability to regulate the synthesis of inflammatory proteins. As a result, inflammation and cortisol are not properly regulated, which can lead to premature contractions and birth. Because depression is a stressor, it probably plays a role in preterm birth. Experts also feel that SGA is linked to depression because it disrupts the neuroendocrine balance. The hormonal end products of hypo or hyperactivity of the hypothalamic-pituitary-adrenal (HPA) axis (i.e., cortisol and norepinephrine) may affect uterine artery blood flow, parturition, and fetal development and growth [9,15]. Effects of depression during pregnancy on fetal outcomes are depicted below in Figure 2.

Furthermore, Fekadu Dadi et al. observed that the adjusted risk of stillbirth was 3.22 times greater among study participants who were depressed during their pregnancy. The path analysis found that prenatal depression had a slight direct effect on stillbirth. However, stillbirth risk was reduced by 73% in research participants who had good stress coping skills [9].

According to Yang et al. (2017), LBW (birth weight less than 2500 gm) is the leading cause of prenatal mortality and morbidity, as well as an increased risk of noncommunicable diseases including diabetes and cardiovascular disease later in life. Low birth weight accounts for more than 15% of all births worldwide, with a higher prevalence in low-income nations. Women with depression and anxiety during pregnancy (DADP), who experienced a preterm birth, had a higher risk of having an LBW baby, according to this study, whereas women with DADP who had a full-term birth did not. In this study, prenatal depression was shown to be prevalent in 18.6% of pregnant women, antenatal anxiety was found to be prevalent in 13.0% of pregnant women, and 9.3% of pregnant women experienced both antenatal depression and anxiety [13]. Therefore, anxiety and depression could be important associations to look for in a pregnant woman.

Hagberg et al. (2018) suggested that women who experience depression during pregnancy have a higher risk of having a child with autism spectrum disorder (ASD) than women who are not affected regardless of antidepressant use. The risk was marginally higher in women with treated depression compared to those with untreated depression; however, the fact that the risk of ASD was not increased in women who were taking antidepressants for other reasons suggests that antidepressants are not responsible. Rather, the findings show that the modest increase in risk associated with antidepressant usage is due to differences in depression intensity. Finally, independent of the existence of anxiety/other psychiatric disorders, the risk of ASD was enhanced among children of mothers with treated or untreated depression; however, the risks were higher among depressed women with comorbid anxiety/psychological disorder [11].

Postpartum Follow-up

According to Al Rawahi et al. (2020), women with prenatal depression have a higher chance of acquiring postnatal depression than women who do not have antenatal depression [17]. A study from the United States indicated that women who had poor mental health during the prenatal period were 11 times more likely to acquire poor mental health during the postnatal period [29]. In another study, 23% of women with postnatal depression said their depressive symptoms began during pregnancy [30]. It was also discovered that women who had previously suffered postnatal depression have a higher probability of experiencing postnatal depression after consecutive deliveries [31].

Ultimately, it was indicated that maternal depression during pregnancy may negatively influence mother-infant health outcomes. To prevent this adverse outcome, depression should be screened, monitored, and managed appropriately when required, keeping risk-benefit in consideration.

Limitation

Our paper shows some limitations: papers in another language were not included and studies published before 2014 were disregarded, thus many valuable studies could have been missed; also, most of the studies, although focused on analyzing both presence and severity of depressive symptoms, do not provide a structured diagnosis of depression in line with the criteria established in the Diagnostic and Statistical Manual of Mental Disorders or the International Classification of Diseases. Some studies did not deploy the same structured approach for gestational length, spontaneous abortion, and birth weight, which are the variables that could be associated with depression during pregnancy, antidepressants, or both. Comparative research on other outcomes, i.e., neonatal adaptation syndrome, longer-term behavior effects, etc., is key areas for further research. Finally, the lack of a control group (i.e., depressed medicated mothers) in some studies greatly reduces the significance of such results.

## Conclusions

We observed higher risks of preterm birth, small for gestational age, stillbirth, low birth weight, and ASD when we took a systematic approach to evaluate the impact of untreated depression on pregnancy, and maternal morbidity can lead to perinatal complication, increased operative delivery, and postpartum depression. Our findings have substantial therapeutic implications for pregnant women and health care professionals because they imply the need for increased surveillance for adverse outcomes in women with untreated depression during pregnancy. Antenatal depression must be detected and treated as soon as possible. Physicians should perform a risk-benefit analysis and carefully detail the risks of untreated depression for both the mother and the fetus once the diagnosis has been verified. Nonpharmacologic therapy for mild-to-moderate depression could be administered first, coupled with a referral to a psychologist if one is available. Psychotherapy alone may not be enough for more critically ill patients, and further antidepressant treatment may be required. To avoid recurrence after treatment has begun, it is vital to evaluate and ensure proper follow-up into the postpartum period.
